# Genome-Wide Analyses and Expression Profiling of *PME*/*PMEI* Gene Families Reveal Their Relevance to Chilling Stress Response and Grafted Healing Efficiency in Cucumber/Pumpkin-Grafted Plants

**DOI:** 10.3390/plants14091294

**Published:** 2025-04-25

**Authors:** Huangfang Lin, Huilan Ye, Qingqing Shao, Saichuan Cheng, Ge Bi, Biying Lin, Honghui Lin, Lei He, Baoying Shen, Haisheng Zhu, Qingfang Wen, Shuang Liu, Qingqing Chen

**Affiliations:** 1 College of Horticulture, Fujian Agriculture and Forestry University, Fuzhou 350002, China; 18060568276@163.com (H.L.); 19835620037@163.com (H.Y.); shaoqingqing807@163.com (Q.S.); 15378306254@163.com (S.C.); 13133094310@163.com (G.B.); linhonghui402@163.com (H.L.); HL15396061256@163.com (L.H.); liushuangsyau@aliyunl.com (S.L.); 000q020062@fafu.edu.cn (Q.C.); 2 Fujian Key Laboratory of Vegetable Genetics and Breeding, Crops Research Institute, Fujian Academy of Agricultural Sciences, Fuzhou 350002, China; zhs0246@163.com (H.Z.); wenqingfang@faas.cn (Q.W.)

**Keywords:** cucumber, pumpkin, *PME*/*PMEI* gene family, graft healing, light intensity mode

## Abstract

Pectin methylesterases (PMEs) and their inhibitors (PMEIs) serve as pivotal enzymes in pectin methylation modifications, playing crucial roles in plant morphogenesis, cell adhesion, and maintenance of the cell wall integrity. However, there have been limited studies exploring the functions of the *PME*/*PMEI* gene families in the healing process of grafted-cucumber seedlings and their responses to stress conditions. In this study, we identified 52 *CsaPME* family members and 33 *CsaPMEI* family members as well as 86 *CmoPME* family members and 36 *CmoPMEI* family members. Comprehensive analyses of the *PME*/*PMEI* gene families in cucumber and pumpkin were conducted using bioinformatics techniques. Additionally, the *PME*/*PMEI* gene families in cucumber and pumpkin exhibited distinct expression modes in different vegetative organs of homologous/heterologous-grafted seedlings and under chilling stress. Notably, the cucumber/pumpkin-grafted seedlings exhibited responses in the roots and leaves involving *PMEI* and type I *proPME* genes, facilitating their adaptation to chilling stress. Additionally, an investigation into the responsiveness of cucumber/pumpkin-grafted seedlings during the healing phase to varying light intensity modes revealed that the implementation of a higher light intensity mode resulted in an upregulation of the expression levels of the majority of *PME/PMEI* family genes, particularly those belonging to the *PME* family, during the critical stages of isolation layer and callus formation. Based on these findings, six key *PME*/*PMEI* family members responsive to different light intensity modes during graft healing were selected. Through the prediction of transcription factor binding sites and an analysis of the response to different light intensity modes during graft healing, four *Dof* transcription factors with potential regulatory relationships with these six key *PME*/*PMEI* genes were identified. This suggests that cucumber/pumpkin-grafted seedlings can regulate key *PME*/*PMEI* genes via *Dof* factors in response to different light intensity modes during the healing process, thereby influencing the progression of graft healing.

## 1. Introduction

The plant cell wall can be morphologically and temporally categorized into three main components: the primary cell wall (PCW) formed during cell growth, the secondary cell wall (SCW) deposited post-growth cessation within the PCW, and the middle lamella (ML) [1]. The PCW primarily comprises pectin, hemicellulose, cellulose, and structural proteins. The SCW mainly consists of hemicellulose, cellulose, and lignin, while the ML is predominantly composed of pectin [2,3]. Pectin polysaccharides possess complex structures and necessitate the coordinated action of various cell wall-modifying enzymes and glycosyltransferases for synthesis from nucleoside sugars within the Golgi apparatus [4,5,6]. The main components of pectin include homogalacturonan (HG), rhamnogalacturonan I (RGI), and rhamnogalacturonan II (RGII) [7]. Pectin plays a crucial role in plant tissue morphogenesis, stress responses, cell adhesion, and maintaining cell wall integrity (CWI) [8,9].

Methylesterification, a crucial chemical modification of pectin molecules, affects their chemical properties and biological functions. This process is regulated by pectin methylesterase (PME) and its inhibitor (PMEI). These enzymes mainly influence the transport of pectin from the Golgi apparatus to the primary cell wall and affect the biomechanical properties of the cell wall including rigidity, elasticity, and permeability [10]. These effects are mediated by the degree and mode of methylesterification [11]. PME (EC: 3.1.1.11) is a member of the carbohydrate esterase family CE8 (http://www.cazy.org/CE8.html) (accessed on 10 June 2024) and has a conserved PME domain at its C-terminus. In higher plants, *PME* genes are classified into type I *proPME* and type II *PME*, based on the presence or absence of an N-terminal pro-domain [12]. The pro-domain of type I *proPME* resembles the conserved PMEI domain found in *PMEI* genes. This domain autoinhibits PME activity, preventing the premature demethylesterification of pectin during protein maturation [13]. Furthermore, the catalysis mode of PME determines the methylesterification mode. In “blockwise” catalysis mode, consecutive methyl ester groups of galacturonic acids are catalyzed, forming a blocky demethylesterified polygalacturonic acid backbone. The produced negatively charged carboxyl groups bind to trivalent metal ions (e.g., Ca^2+^ and Al^3+^), forming an egg-crate structure that hardens the cell wall into a rigid gel [14]. In contrast, in “random” catalysis mode, demethylesterified groups are randomly distributed along the polygalacturonic acid backbone. This arrangement increases the binding sites for pectinolytic enzymes, thereby facilitating the depolymerization of pectin into galacturonic acid, leading to softening and relaxation of the cell wall [15]. The degree of methylesterification, determined by the proportion of methylesterified galacturonic acid residues, is influenced by both PME and PMEI. During their synergistic action, PMEI binds to the active site of PME, forming a stoichiometric non-covalent 1:1 complex under varying pH conditions [16,17]. This complex inhibits PME activity. This process can slow down PME-induced demethylesterification and promote the release of protons, leading to cell wall-degrading enzyme-mediated depolymerization. Consequently, this results in cell wall loosening and expansion [18,19].

Currently, 66, 41, 42, 43, 70, 41, and 64 *PME* genes as well as 71, 35, 37, 49, 49, 8, and 39 *PMEI* genes have been identified in the genomes of *Arabidopsis thaliana*, *Oryza sativa*, *Sorghum bicolor*, *Zea mays*, *Populus trichocarpa*, *Vitis vinifera*, and *Solanum lycopersicum*, respectively [20,21,22]. Researchers have conducted functional studies on *PME* and *PMEI* genes, examining their roles in seed germination, root and shoot elongation, fertility, stress resistance, and graft union healing [23,24]. For example, the *AtPME3-1* loss-of-function mutant exhibits advanced seed germination, decreased root hair formation, and a downregulated expression of genes related to gibberellic acid (GA) and lipid metabolism, suggesting *AtPME3*’s involvement in multiple growth and development processes [25]. Furthermore, the antisense expression of *BoPMEI1* in *Arabidopsis* results in the inhibition of pollen tube growth and male sterility, while *AtPMEI10*, *AtPMEI11*, and *AtPMEI12* contribute to resistance against *Botrytis cinerea* [26]. Overexpression of *AtPMEI5* activates Dof transcription factors, thereby promoting graft union healing [27].

Cucumbers (*Cucumis sativus* L.) and pumpkins (*Cucurbita moschata* Duchesne ex Poir.) are two significant cucurbit crops, widely distributed, in high demand, and possess promising market potential. To enhance stress resistance, mitigate continuous cropping challenges, and substantially improve the yield and quality in cucumber production, pumpkin is frequently used as the rootstock, with cucumber serving as the scion for grafting [28]. This method is easy to operate and features low production costs. In agriculturally sophisticated nations like Japan and Germany, the utilization of grafted-cucumber seedlings has surpassed a 90% adoption rate [29]. Conversely, in China, the current application rate stands at merely 30% [30], primarily confined to protected cultivation systems. This disparity underscores the vast untapped potential for cucumber grafting technology. Additionally, prior research has shown that variations in light intensity impact the efficiency of graft healing. Nevertheless, as of now, there have been no investigations into the effects of differing light intensities on *PME*/*PMEI* gene family members during the graft healing process in cucumber and pumpkin. Consequently, utilizing the genomes of cucumbers and pumpkins, this study analyzed and characterized the *PME*/*PMEI* gene families in both species. By accessing the NCBI public database and cucurbitaceae database, we examined the expression modes of *PME*/*PMEI* gene families in different vegetative organs of homologous- and heterologous-grafted seedlings as well as their response to abiotic stress. Additionally, using the transcriptome sequencing data from our research, we examined the mechanisms by which the *PME*/*PMEI* gene families respond to varying light intensity modes during cucumber and pumpkin graft healing. The objective of this study was to clarify the biological roles and underlying mechanisms of the *PME*/*PMEI* gene families in cucumber and pumpkin, specifically in relation to growth, development, stress resistance, and the graft healing process in grafted seedlings. Our findings are pivotal for gaining insights into the mechanisms of graft healing and stress response in cucumber- and pumpkin-grafted plants and offer candidate genes for the development of future cultivars with improved graft healing efficiency and stress tolerance.

## 2. Results

### 2.1. Identification, Chromosomal Localization, and Physicochemical Property Analysis of PME/PMEI Gene Families in Cucumber and Pumpkin

**Figure 1:**
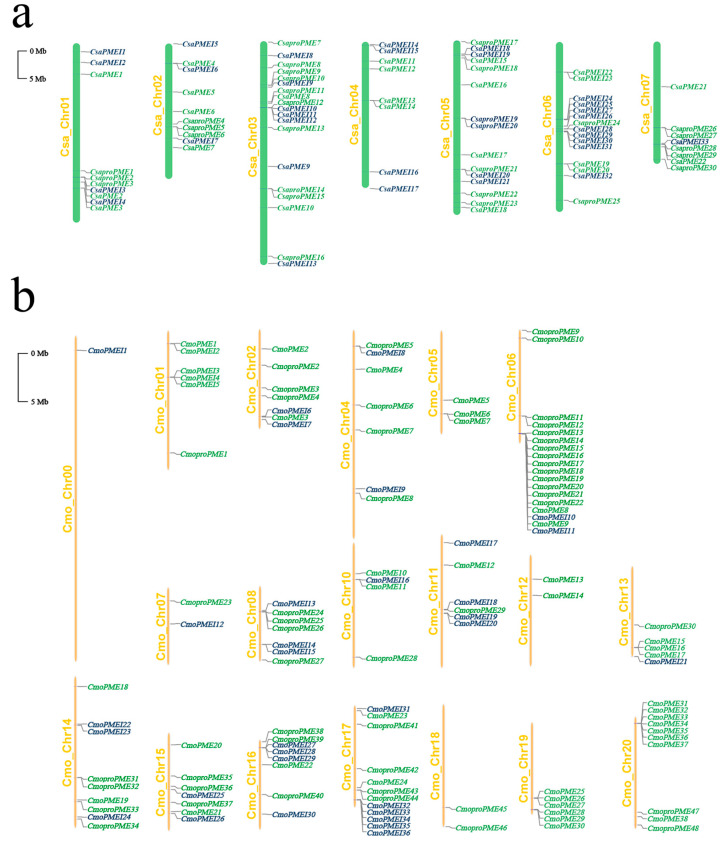
Chromosomal localization of *PME*/*PMEI* family members in (**a**) cucumber and (**b**) pumpkin. The genes in green font belong to the *PME* family, and the genes in dark blue font belong to the *PMEI* family.

Following screening and validation, we identified 52 members of the *CsaPME* family including 30 type I *CsaproPMEs* and 22 type II *CsaPMEs* as well as 33 members of the *CsaPMEI* family in the cucumber genome. Similarly, in the pumpkin genome, we identified 86 members of the *CmoPME* family, comprising 48 type I *CmoproPMEs* and 38 type II *CmoPMEs*, alongside 36 members of the *CmoPMEI* family. Subsequently, these genes were renamed based on their chromosomal locations (Figure 1a,b). In cucumber, Chr3 is the longest chromosome and harbors the highest number of *CsaPME* family members (14, 26.9%) while Chr6 contains the most *CsaPMEI* family members (11, 33.3%). In pumpkin, Chr0 is the longest chromosome but harbors only one member of the *CmoPMEI* family, *CmoPMEI1*. Furthermore, Chr18-20 exclusively harbor members of the *CmoPME* family.

To investigate the characteristics of the *PME/PMEI* family members in cucumber and pumpkin, we conducted an analysis of their physicochemical properties. According to Table S1, the amino acid counts for the cucumber genes *CsaproPMEs*, *CsaPMEs*, and *CsaPMEIs* ranged from 490 (*CsaProPME9*) to 605 (*CsaProPME13*), 94 (*CsaPME14*) to 409 (*CsaPME10*), and 147 (*CsaPMEI8*) to 673 (*CsaPMEI6*), respectively. The molecular weights correspondingly ranged from 54.43 kDa (*CsaProPME9*) to 66.39 kDa (*CsaProPME13*), 10.08 kDa (*CsaPME14*) to 45.06 kDa (*CsaPME10*), and 15.90 kDa (*CsaPMEI8*) to 75.31 kDa (*CsaPMEI6*). For *CsaproPMEs*, *CsaPMEs*, and *CsaPMEIs*, there were 22, 10, and 18 basic proteins; 5, 1, and 14 unstable proteins; and 16, 12, and 25 proteins with signal peptides, respectively. Furthermore, 15 *CsaPMEIs* exhibited hydrophobicity, whereas *CsaPMEs* and the remaining *CsaPMEIs* were hydrophilic. Similarly, in pumpkin, the amino acid counts for *CmoproPMEs*, *CmoPMEs*, and *CmoPMEIs* ranged from 232 (*CmoProPME39*) to 2335 (*CmoProPME10*), 115 (*CmoPME17*) to 1259 (*CmoPME18*), and 138 (*CmoPMEI35*) to 986 (*CmoPMEI32*), respectively. The molecular weights correspondingly ranged from 25.91 kDa (*CmoProPME39*) to 262.13 kDa (*CmoProPME10*), 12.50 kDa (*CmoPME17*) to 139.20 kDa (*CmoPME18*), and 14.75 kDa (*CmoPMEI35*) to 111.14 kDa (*CmoPMEI32*). The *CmoproPMEs*, *CmoPMEs*, and *CmoPMEIs* contained 31, 22, and 14 basic proteins as well as 9, 7, and 16 unstable proteins, 2, 4, and 11 hydrophobic proteins, and 23, 19, and 24 proteins with signal peptides, respectively. Additionally, the majority of *PME*/*PMEI* family members in cucumber and pumpkin were found in the chloroplasts and cell walls.

### 2.2. Phylogenetic Evolution Analysis of the PME/PMEI Gene Families

**Figure 2:**
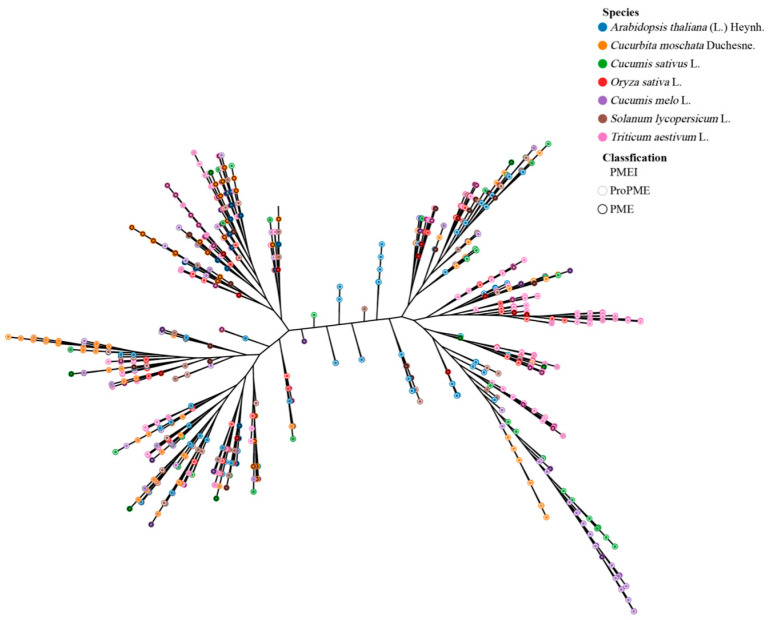
Phylogenetic relationships of the *PME*/*PMEI* families across seven species.

To investigate the biological characteristics and genetic relationships of the *PME*/*PMEI* family across various species, we used MEGA11 software (version 11.0.13) to select amino acid sequences of the *PME*/*PMEI* family members from *Arabidopsis thaliana* (43 *proPMEs*, 23 *PMEs*, and 71 *PMEIs*), *Oryza sativa* (18 *proPMEs*, 23 *PMEs*, 35 *PMEIs*), *Triticum aestivum* (49 *proPMEs*, 78 *PMEs*, 144 *PMEIs*), *Solanum lycopersicum* (36 *proPMEs*, 28 *PMEs*, 39 *PMEIs*), *Cucumis melo* (30 *proPMEs*, 26 *PMEs*, 62 *PMEIs*) as well as *Cucumis sativus* and *Cucurbita moschata* for multiple sequence alignment and the construction of a phylogenetic evolutionary tree (Figure 2). The analysis revealed that the evolutionary tree can be divided into three clades, with distinct differences among the *proPME*, *PME*, and *PMEI* genes and slighter differences between *proPME* and some *PME* genes. From an evolutionary perspective, the *PME/PMEI* family members of *Oryza sativa*—*Triticum aestivum* and *Cucumis* species (*Cucumis sativus*, *Cucurbita moschata*, *Cucumis melo*)—exhibit close phylogenetic relationships, respectively, which indicates similar botanical functions and a degree of evolutionary conservation. Taxonomically, there were notable distinctions among the *PME*/*PMEI* family members of Cucurbitaceae, Solanaceae, Brassicaceae, and Poaceae, suggesting the development of unique genetic traits during evolutionary processes.

### 2.3. Gene Structure, Conserved Motifs, and Cis-Acting Elements of the PME/PMEI Families in Cucumber and Pumpkin

**Figure 3:**
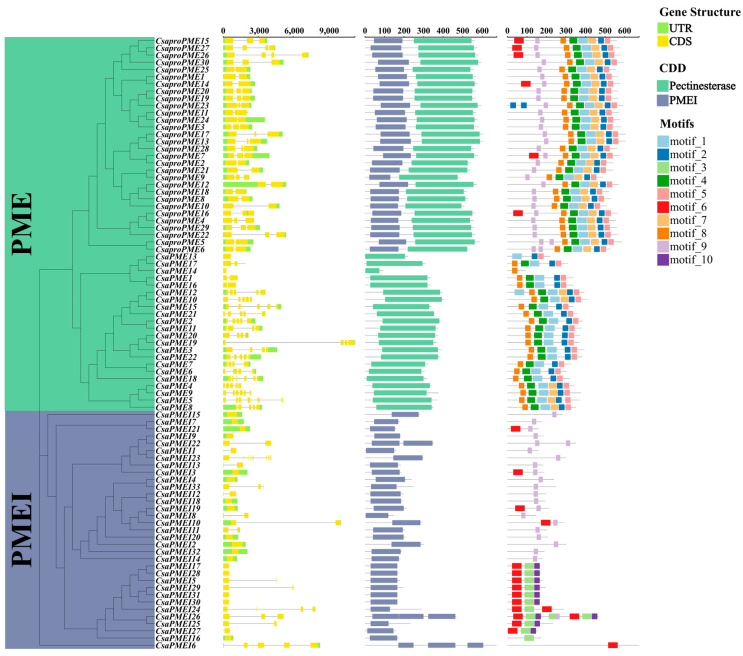
Analysis of the phylogenetic trees, gene structures, conserved domains, and conserved motifs of *PME*/*PMEI* families in cucumber.

**Figure 4:**
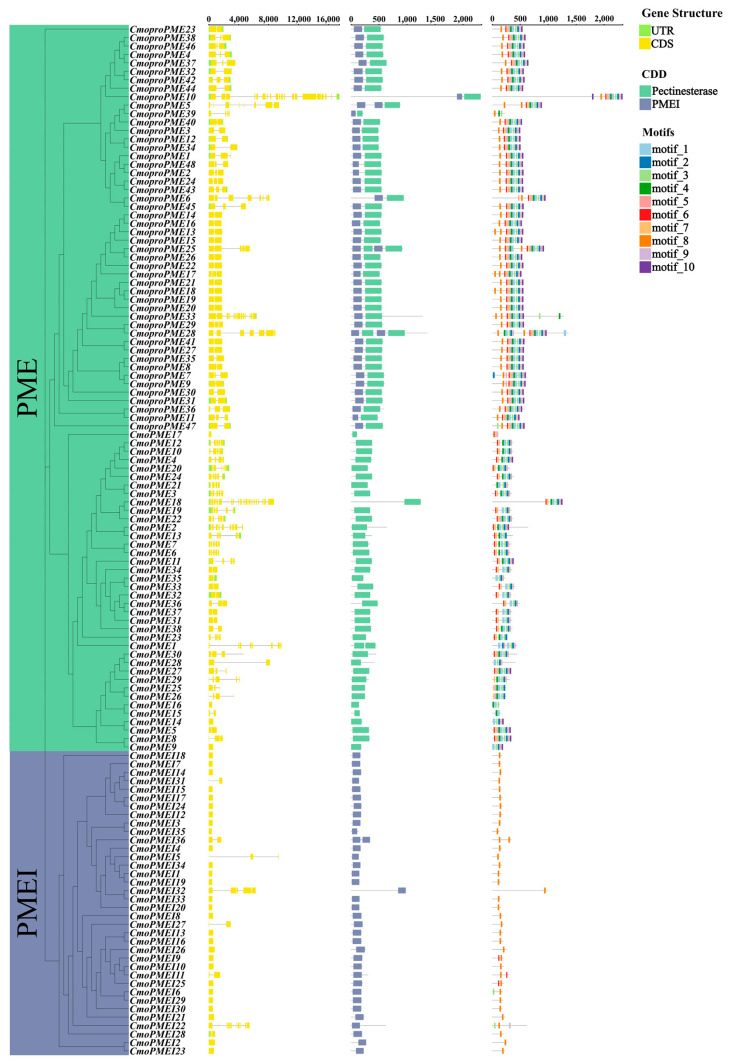
Analysis of the phylogenetic trees, gene structures, conserved domains, and conserved motifs of *PME*/*PMEI* families in pumpkin.

This study aimed to delve deeper into the gene structures of the *PME*/*PMEI* family members in cucumber and pumpkin by examining their phylogenetic relationships, exon-intron organizations, conserved motifs, and architectural features (Figure 3 and Figure 4). The results demonstrated that the *PME*/*PMEI* family in cucumber and pumpkin could be categorized into three groups: *proPME*, *PME*, and *PMEI*. This classification aligns with the phylogenetic relationships observed among the *PME*/*PMEI* families of *Arabidopsis*, *Oryza sativa*, *Triticum aestivum*, *Solanum lycopersicum*, *Cucumis melo*, *Cucumis sativus*, and *Cucurbita moschata*.

The analysis of exon-intron structures showed that the average exon numbers for *CsaproPMEs*, *CsaPMEs*, and *CsaPMEIs* were 2.43 (predominantly 1–2), 2.91 (predominantly 1–4), and 2.94 (predominantly 1–4), respectively. In contrast, *CmoproPMEs*, *CmoPMEs*, and *CmoPMEIs* exhibited average exon numbers of 3.46 (primarily 2–3), 4.18 (primarily 2–5), and 1.58 (solely 1), respectively. Additionally, the analysis of conserved protein domains revealed that type II *PMEs* and *PMEIs* in cucumber and pumpkin possessed conserved pectinesterase and PMEI domains, respectively, whereas type I *proPMEs* harbored both conserved domains.

MEME was used to identify conserved protein motifs in the *PME*/*PMEI* families of cucumber and pumpkin. The results revealed significant conservation of motifs 1, 2, 4, 5, and 8 in both *CsaproPMEs* and *CsaPMEs*. Notably, *CsaproPMEs* also harbored motif 7, which was found in only five *CsaPMEs*. Furthermore, *CsaproPMEs* and *CsaPMEIs* exhibited shared motifs 6 and 9, whereas *CsaPMEIs* uniquely possessed motifs 3 and 10, distinguishing them among type I *proPMEs*, type II *PMEs*, and *PMEIs*. Motifs 1–7 were conserved in *CmoproPMEs* and *CmoPMEs*, with *CmoproPMEs* additionally containing motifs 9 and 10. Among the *CmoPMEs*, eight members harbored motif 9, and nine harbored motif 10. Furthermore, *CmoproPMEs* and *CmoPMEIs* exhibited shared motif 8. These findings indicate a closer phylogenetic relationship between type I *proPMEs* and type II *PMEs*, with motif 8 serving as a pivotal factor in distinguishing type I *proPMEs* and *PMEIs* from type II *PMEs*. Moreover, except for individual genes, the conserved motif structures of the cucumber and pumpkin *PME* families demonstrated higher conservation compared with the *PMEI* families.

**Figure 5:**
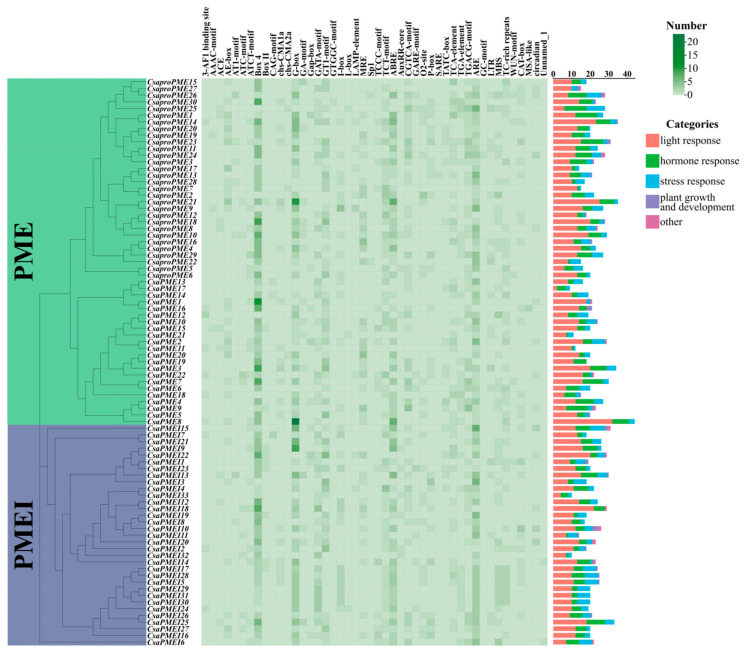
Analysis of cis-acting elements of *PME*/*PMEI* families in cucumber.

**Figure 6:**
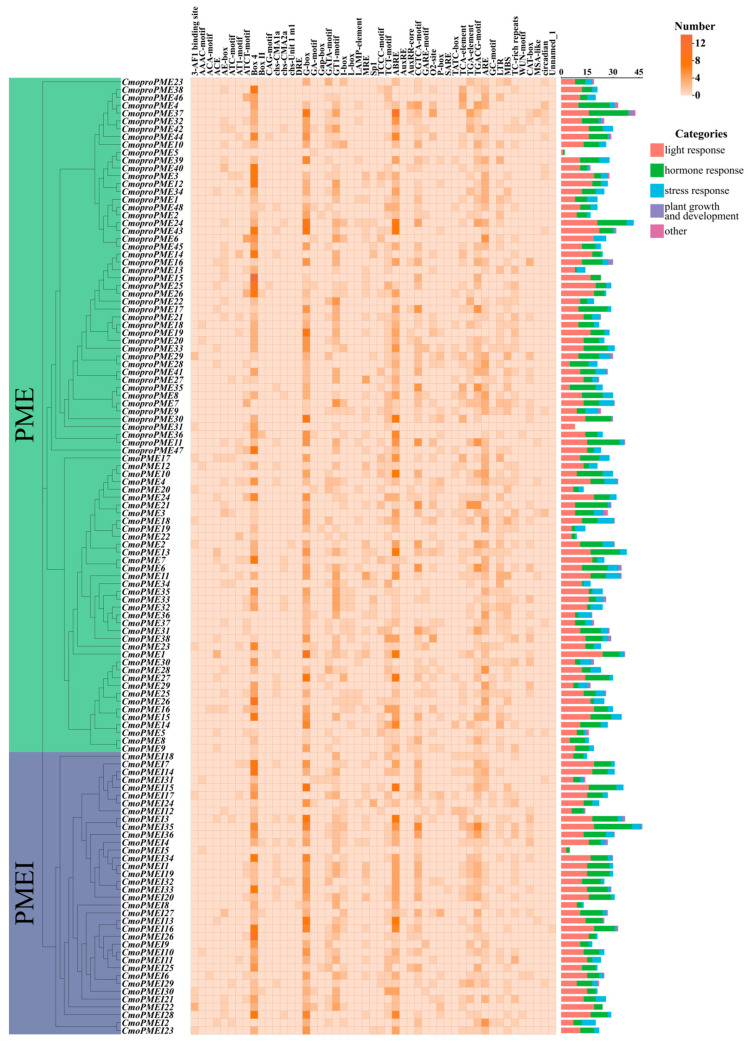
Analysis of cis-acting elements of *PME*/*PMEI* families in pumpkin.

To delve deeply into the functional differences among the promoters of the *PME*/*PMEI* family members in cucumber and pumpkin, we extracted the promoter region sequences located 2000 bp upstream of the start codons of these family members for cis-acting element analysis (Figure 5 and Figure 6). The results indicated that the identified cis-acting elements primarily involved functions related to light, hormone, stress responses, and plant growth and development. In cucumber and pumpkin, 46 and 49 cis-acting elements were identified, respectively, with 45 common elements shared between them. These common elements encompassed 24 types of light-responsive elements, 11 hormone-responsive elements, 6 stress-responsive elements, 2 growth and development-related elements, 1 photoperiod-responsive element, and 1 phytochrome degradation-related element. Among these, cucumber uniquely possessed one light-responsive element (GTGGC-motif), while pumpkin had three exclusive light-responsive elements (ACA-motif, chs-Unit 1 m1, DRE) and one hormone-responsive element (AuxRE). Notably, both the cucumber and pumpkin *PME*/*PMEI* family members abundantly possessed light-responsive elements such as Box 4, G-box, and GT1-motif as well as one abscisic acid-responsive element (ABRE) and one anaerobiosis-responsive element (ARE). Additionally, pumpkin specifically harbored a high abundance of the light-responsive element TCT-motif and two methyl jasmonate (MeJA)-responsive elements (CGTCA-motif and TGACG-motif). These findings suggest that the *PME/PMEI* genes in cucumber and pumpkin exhibit a degree of conservation during evolution while playing crucial roles in the light, hormone, and stress response networks.

### 2.4. Collinearity and Selection Pressure Analysis of the PME/PMEI Families

**Figure 7:**
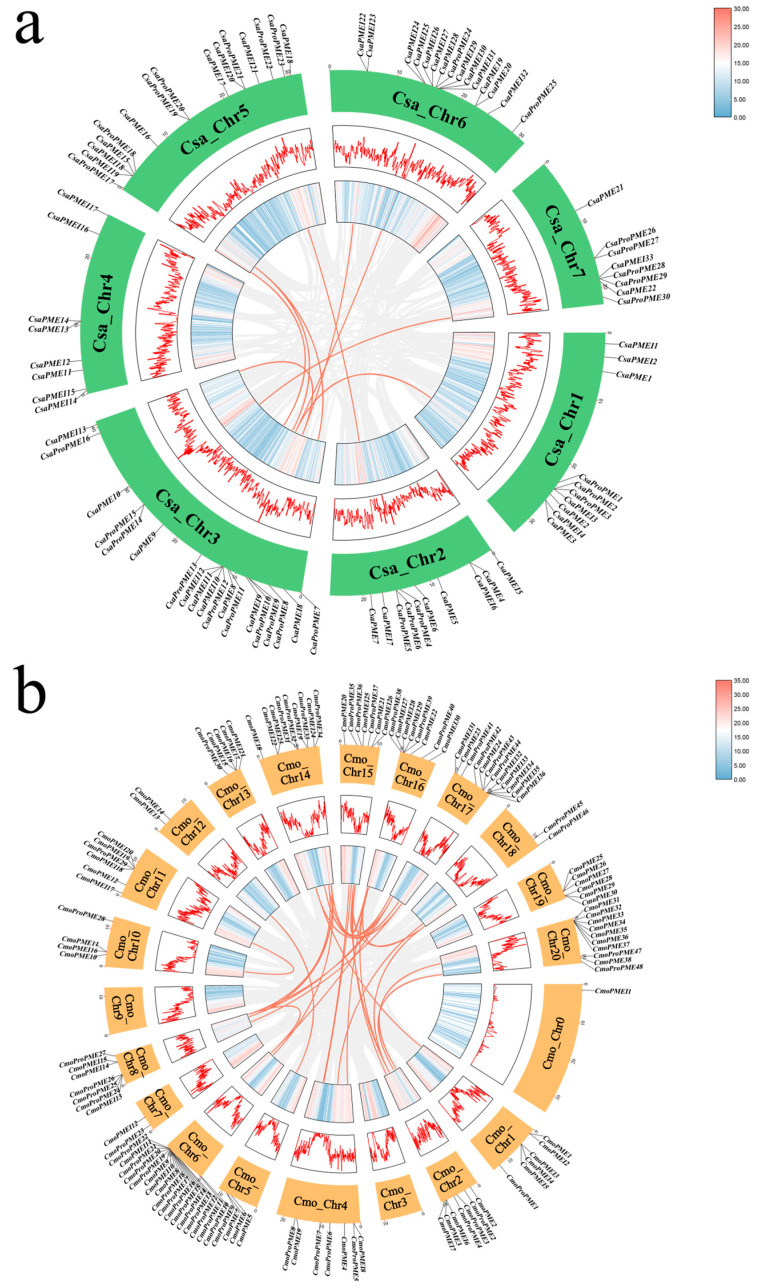
Collinearity analysis of *PME/PMEI* families in cucumber and pumpkin. (**a**) Collinearity analysis of *PME*/*PMEI* family members in cucumber. (**b**) Collinearity analysis of *PME*/*PMEI* family members in pumpkin.

**Figure 8:**
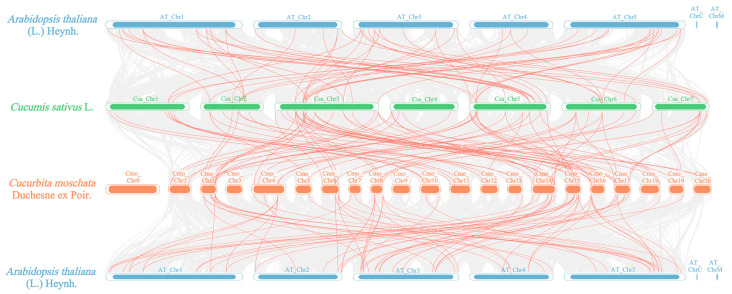
Collinearity analysis of the *PME*/*PMEI* families in cucumber, pumpkin, and *Arabidopsis*.

Figure 7a illustrates that within the *PME*/*PMEI* families of cucumber, one pair of tandem duplication events (*CsaPMEI9*/*CsaPMEI13*) and eight pairs of segmental duplication events existed compared with 28 pairs of segmental duplication events observed in pumpkin (Figure 7b). These findings suggest that the expansion of the *PME*/*PMEI* gene families in cucumber and pumpkin is primarily due to chromosomal segmental duplications. To further elucidate the evolutionary mechanisms of the *PME*/*PMEI* gene families in cucumber and pumpkin, we employed MCScanX to perform a collinearity analysis of their family members with those in *Arabidopsis* (Figure 8). The analysis revealed 85 collinear pairs between 51 *PME*/*PMEI* genes in cucumber and 67 in pumpkin, 57 collinear pairs between 34 in cucumber and 46 in Arabidopsis, and 65 collinear pairs between 122 in pumpkin and 40 in Arabidopsis.

**Figure 9:**
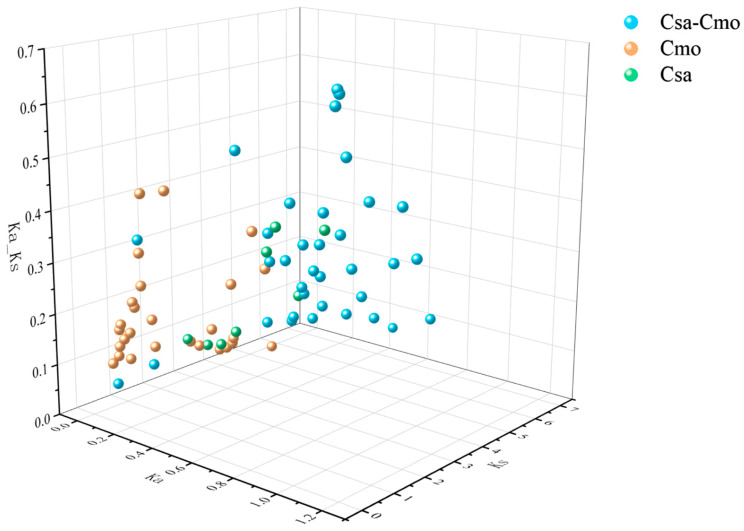
Ka/Ks analysis of duplication events and collinear gene pairs between cucumber and pumpkin.

To investigate the selection pressure and evolutionary rates of the *PME*/*PMEI* families in cucumber and pumpkin, we calculated the Ka (non-synonymous substitution rate), Ks (synonymous substitution rate), and Ka/Ks ratios for duplication events within and between these species (Figure 9). After excluding gene pairs with significant sequence divergences, our results showed that the Ka/Ks ratios for all gene pairs were less than 1, indicating purifying selection pressure during evolution.

### 2.5. GO Enrichment and Protein–Protein Interaction Network Analysis of PME/PMEI Gene Families in Cucumber and Pumpkin

**Figure 10:**
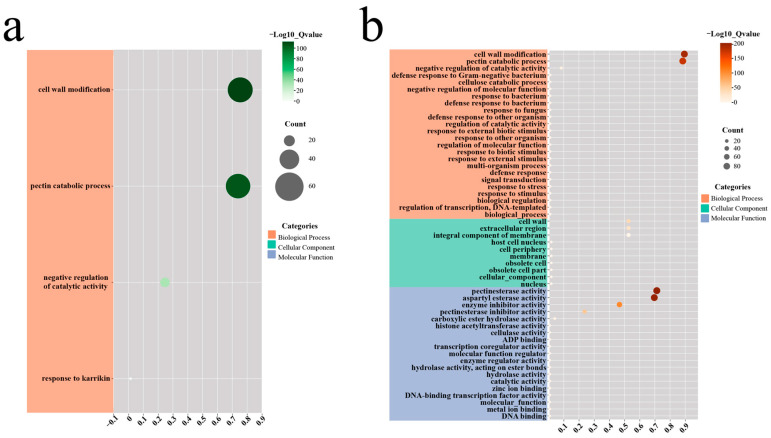
GO enrichment analysis of the *PME*/*PMEI* gene family members in (**a**) cucumber and (**b**) pumpkin.

To gain deeper insights into the functional roles and regulatory mechanisms of *PME*/*PMEI* gene families in cucumber and pumpkin, we performed GO enrichment analysis and protein–protein interaction (PPI) network analysis. The GO classifications of *PME*/*PMEI* genes in both cucumber and pumpkin were predominantly enriched in biological processes related to cell wall modification, pectin catabolism, and negative regulation of catalytic activity (Figure 10a,b). Furthermore, the pumpkin *PME*/*PMEI* genes were also enriched in stress response-related processes. However, the cucumber *PME*/*PMEI* genes showed no enrichment in the molecular function and cellular component categories of the GO classifications. In contrast, the pumpkin *PME*/*PMEI* genes were enriched in various molecular functions including pectin lyase activity, aspartyl esterase activity, and enzyme activity as well as in cellular components such as the cell wall, extracellular region, and cell membrane.

**Figure 11:**
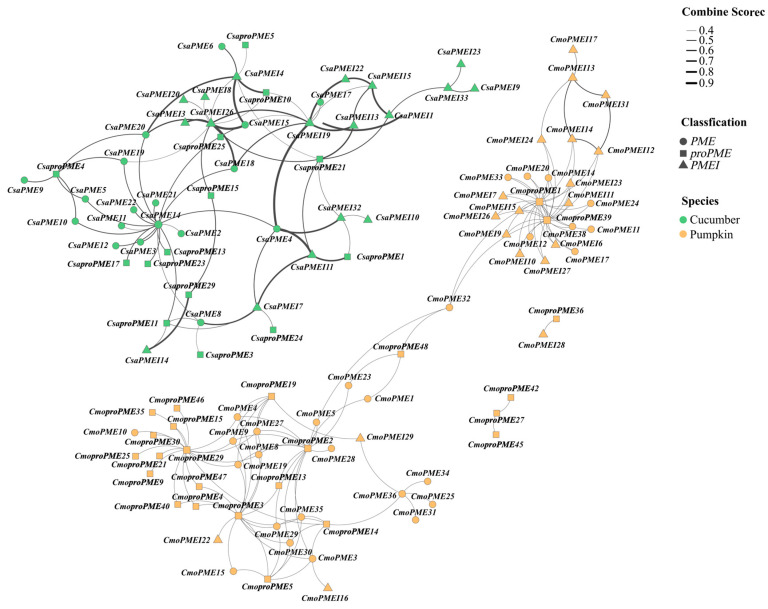
Protein–protein interaction (PPI) network analysis of the *PME*/*PMEI* gene families in cucumber and pumpkin.

Additionally, PPI prediction results revealed interactions among members within and between the *PMEIs* and *PMEs* in both cucumber and pumpkin (Figure 11). In the cucumber PPI network, strong interactions were observed between *CsaPMEIs* and *CsaPMEs*, while interactions among *CsaPMEs* were relatively weaker. Notably, *CsaPME14* and *CsaPMEI26* interacted extensively with other members of the *PME*/*PMEI* family in cucumber. In contrast, robust interactions were detected among *CmoPMEIs* in the pumpkin PPI network. Furthermore, *CmoproPMEs*, including *CmoproPME1*, *CmoproPME2*, *CmoproPME3*, *CmoproPME29*, and *CmoproPME39*, exhibited extensive interactions with other members within the *PME/PMEI* family of pumpkin proteins.

### 2.6. Prediction of Regulatory Relationships Between Transcription Factors and the PME/PMEI Gene Families in Cucumber and Pumpkin

**Figure 12:**
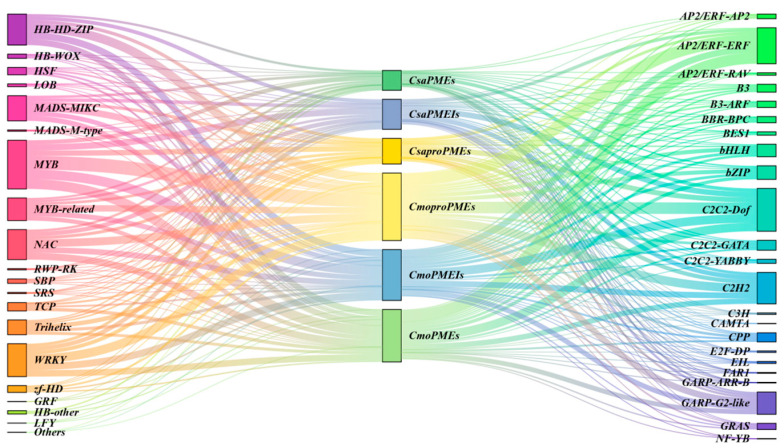
Predicted regulatory network between the transcription factors and *PME*/*PMEI* gene families in cucumber and pumpkin.

Regulatory interactions between transcription factors (TFs) and the *PME*/*PMEI* gene families in cucumber and pumpkin were examined using the Fimo online platform. The results revealed that a greater number of TFs were implicated in regulating *CmoproPMEs* and *CsaPMEIs* in both species (Figure 12). Specifically, 606 TFs formed 3973 regulatory interactions with 33 *CsaPMEIs*, whereas 954 TFs established 37 regulatory interactions with 48 *CmoproPMEs*. Furthermore, several TF families, such as ERF, Dof, C2H2, WRKY, HD-ZIP, MYB, and NAC, were involved in regulating the *PME*/*PMEI* families in both species. These results imply that the *PME*/*PMEI* families are predominantly regulated by TFs related to growth and development, stress responses, and ethylene signaling pathways.

### 2.7. Analysis of Expression Characteristics of the PME/PMEI Gene Family in Cucumber/Pumpkin-Grafted Seedlings

#### 2.7.1. Expression Analysis of the *PME/PMEI* Gene Families Across Different Vegetative Organs and in Response to Chilling Stress in Homografted and Heterografted Cucumber and Pumpkin Seedlings

**Figure 13:**
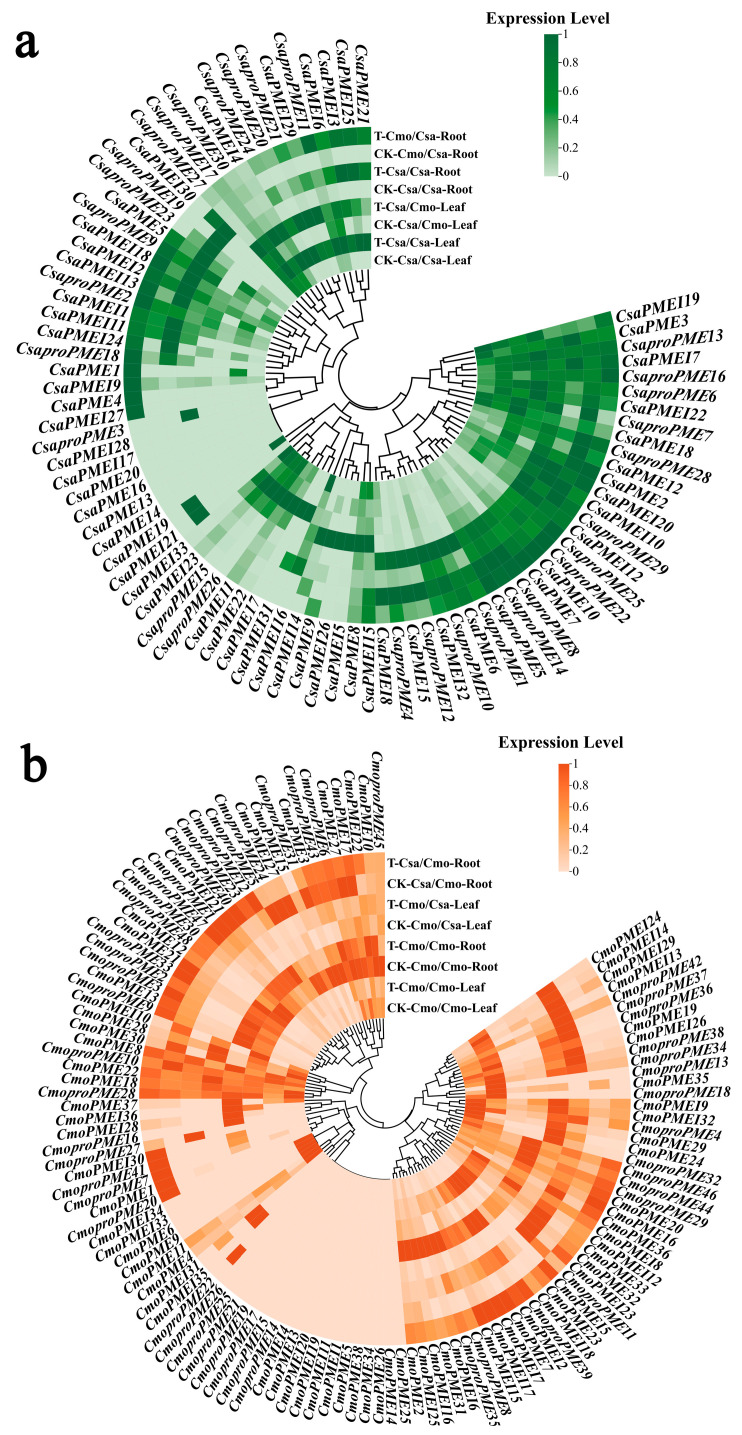
Expression analysis of the *PME*/*PMEI* gene families in different vegetative organs and under chilling stress in the homografted and heterografted (**a**) cucumber and (**b**) pumpkin seedlings. In this figure, CK represents the conventional temperature treatment, while T represents the chilling stress treatment.

To investigate the expression profiles of the *PME/PMEI* gene family in different organs of cucumber- and pumpkin-grafted seedlings (both homografts and heterografts) under chilling stress, differentially expressed gene (DEG) screening was conducted based on public transcriptome data using a *p*-value < 0.05 and a fold change (FC) ≥ 1.2 as the criterion (Figure 13a,b). Under the conventional temperature treatment, among the various cucumber-grafted seedling combinations, the expression levels of five PME/PMEI family members were higher in the roots of the cucumber rootstocks than in the true leaves of the cucumber scions, primarily consisting of *CsaproPMEs*, while the expression levels of three *PME*/*PMEI* family members were lower in the roots of the cucumber rootstocks than in the true leaves of the cucumber scions. Additionally, the expression levels of four *PME*/*PMEI* family members were only higher in the roots of the cucumber rootstocks compared with the true leaves of the cucumber scions in heterograft combinations; one *CsaproPME* gene showed higher expression in the true leaves of the heterograft scions than in the homograft scions. In the various pumpkin-grafted seedling combinations, the expression levels of five and two *PME*/*PMEI* family members were higher and lower, respectively, in the roots of the pumpkin rootstocks compared with the true leaves of the pumpkin scions. Furthermore, the expression levels of three and two *PME*/*PMEI* family members were higher and lower, respectively, only in the roots and true leaves of the heterograft seedlings.

Under chilling stress treatment, there were three, nine, three, and nine *PME*/*PMEI* family members with expression levels higher than those under normal temperature treatment in the true leaves of homograft scions, the roots of the homograft rootstocks, the true leaves of the heterograft scions, and the roots of the heterograft rootstocks of cucumber, respectively. Correspondingly, one, five, two, and two family members showed lower expression levels. Additionally, under chilling stress treatment, three and one *PME*/*PMEI* family members exhibited higher expression levels in the true leaves and roots of the cucumber heterografts, respectively, compared with their homograft counterparts. For pumpkin, there were four, seventeen, six, and fifteen *PME*/*PMEI* family members with expression levels higher than those under normal temperature treatment in the true leaves of the homograft scions, the roots of the homograft rootstocks, the true leaves of the heterograft scions, and the roots of the heterograft rootstocks, respectively, while, three, five, two, and five family members showed lower expression levels. Furthermore, under chilling stress, two *PME*/*PMEI* family members exhibited higher expression levels in the roots of the pumpkin heterograft rootstocks compared with their homograft counterparts.

#### 2.7.2. Expression Analysis of PME/PMEI Gene Family in Response to Different Light Intensity Modes During the Graft-Healing Phase of Cucumber/Pumpkin-Grafted Seedlings

**Figure 14:**
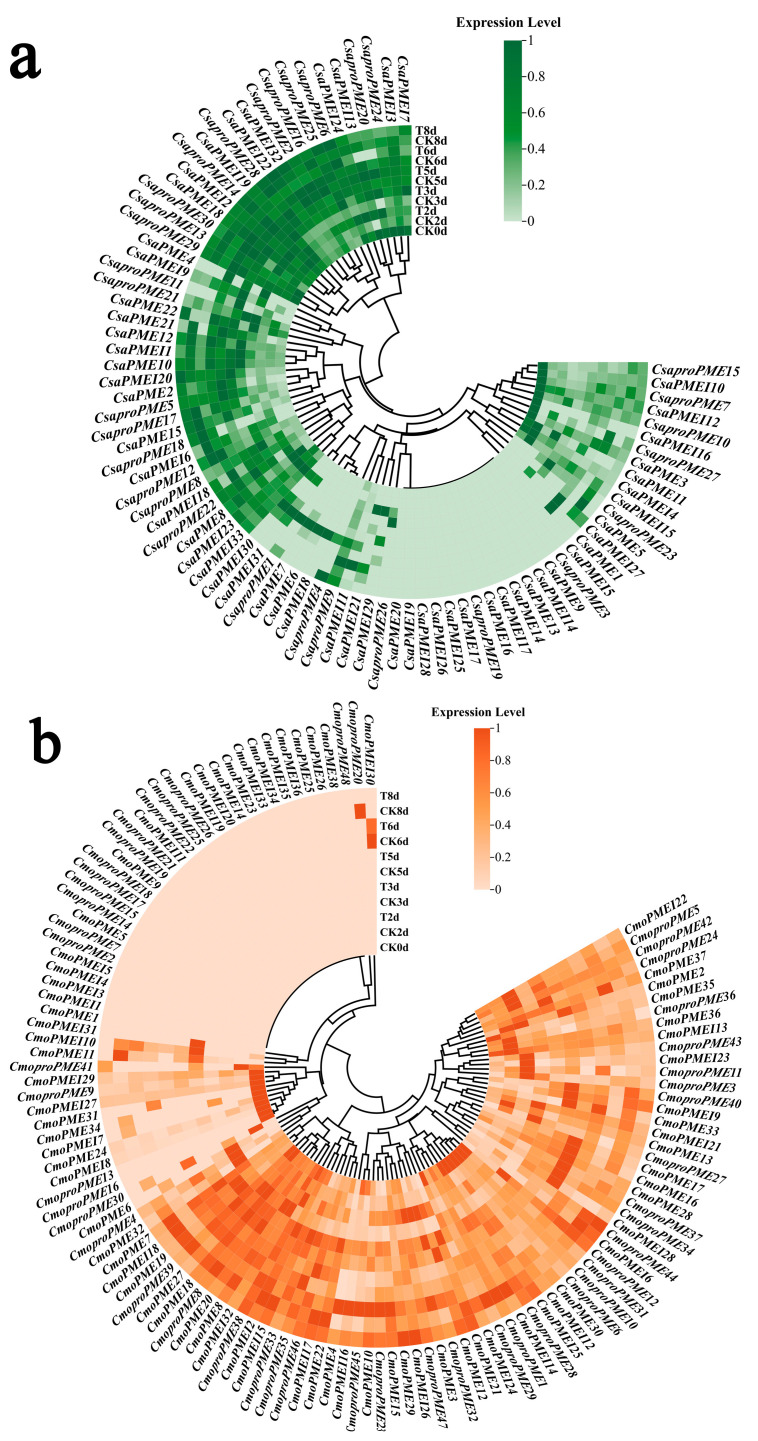
Analysis of (**a**) cucumber and (**b**) pumpkin *PME*/*PMEI* gene family expression in response to different light intensity modes during the graft-healing phase of cucumber/pumpkin-grafted seedlings.

To investigate the response mechanism of the *PME/PMEI* gene family to different light intensity modes during the graft-healing phase of the cucumber/pumpkin-grafted seedlings, DEGs were screened based on the transcriptome sequencing results using the criterion of fold change ≥ 1.2 and *p*-value < 0.05 for analysis (Figure 14a,b). During the isolation layer formation stage, compared with CK_2d_, we observed significant upregulation of eighteen *CmoPME*, four *CmoPMEI*, six *CsaPME*, and three *CsaPMEI* family members under T_2d_. Conversely, three *CmoPME*, three *CmoPMEI*, three *CsaPME*, and one *CsaPMEI* family members were significantly downregulated. Similarly, during the callus formation stage, compared with CK_5d_, eleven *CmoPME*, two *CmoPMEI*, thirteen *CsaPME*, and two *CsaPMEI* family members were upregulated under T_5d_. In contrast, fourteen *CmoPME*, five *CmoPMEI*, four *CsaPME*, and four *CsaPMEI* family members exhibited downregulation. During the vascular bundle formation stage, compared with CK_8d_, sixteen *CmoPME*, seven *CmoPMEI*, seven *CsaPME*, and nine *CsaPMEI* family members were upregulated at T_8d_. Conversely, nine *CmoPME*, three *CmoPMEI*, four *CsaPME*, and one *CsaPMEI* family members were downregulated.

These results indicate that during different stages of cucumber/pumpkin graft healing, the *PME* and *PMEI* families exhibit differential responses to varying light intensity modes. Notably, type I *proPMEs* and *PMEIs* remained active throughout the various grafting healing stages.

### 2.8. Identification and Related Analyses of the Key Members of the PME/PMEI Gene Families in Cucumber and Pumpkin

We analyzed the expression modes of the *PME/PMEI* gene families in different vegetative organs of various grafting combinations of cucumber and pumpkin under both the conventional temperature treatment and chilling stress treatment. Additionally, we investigated the temporal response characteristics of the *PME*/*PMEI* gene families to different light intensity modes during the grafting healing process of the cucumber/pumpkin-grafted seedlings. The results revealed specific expression modes of *CsaPME15*, *CsaproPME10*, *CsaPMEI19*, *CmoPME17*, *CmoproPME39*, and *CmoPMEI27* during the growth and development of the grafted seedlings in response to chilling stress and in the light response process during graft healing. Based on these findings, we speculate that these six genes may have unique functions.

#### 2.8.1. Structural Analysis of Proteins Encoded by Key Members of the PME/PMEI Gene Families in Cucumber and Pumpkin

**Figure 15:**
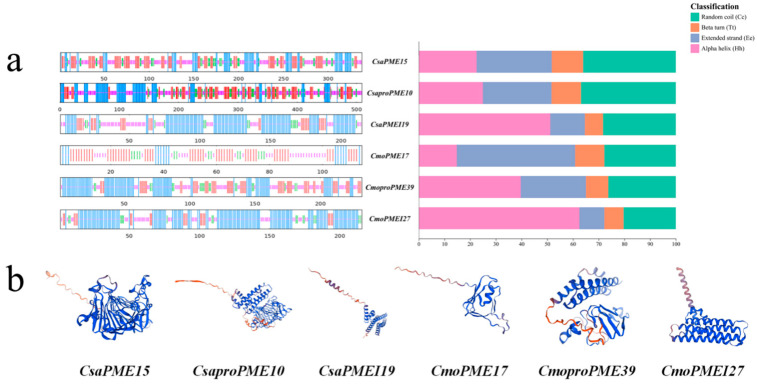
Spatial structure analysis of proteins encoded by key members of the *PME*/*PMEI* gene families in cucumber and pumpkin. (**a**) Secondary structure analysis. (**b**) Tertiary structure analysis.

The spatial structures of six key proteins within the *PME*/*PMEI* gene families of cucumber and pumpkin were examined (Figure 15). The study revealed that *CsaPME15*, *CsaproPME10*, *CsaPMEI19*, *CmoPME17*, *CmoproPME39* and *CmoPMEI27* were modeled on A0A6J1E7N2.1.A, A0A5D3DJL7.1.A, A0A6J1E526.1.A, A0A6J1E808.1.A, A0A1S3AVW4.1.A, and A0A5A7TEM1.1.A, respectively, and the structures of the key proteins of type I *proPMEs*, type II *PMEs*, and *PMEIs* were mainly composed of α-helices and random coils, with some variations. Notably, *CsaPMEI19* and *CmoPMEI27* exhibited a higher proportion of α-helices (51.16% and 62.50%, respectively) compared with the other four *PME* gene family members. Furthermore, the main difference between type I *proPMEs* and type II *PMEs* lies in the proportion of α-helices and extended strand structures. This difference was particularly evident in *CmoPME17* and *CmoproPME39*, which had 14.78% and 39.66% α-helices and 46.09% and 25.43% extended strand structures, respectively.

#### 2.8.2. Construction of the Regulatory Network for Key Members of the PME/PMEI Gene Families in Cucumber and Pumpkin

**Figure 16:**
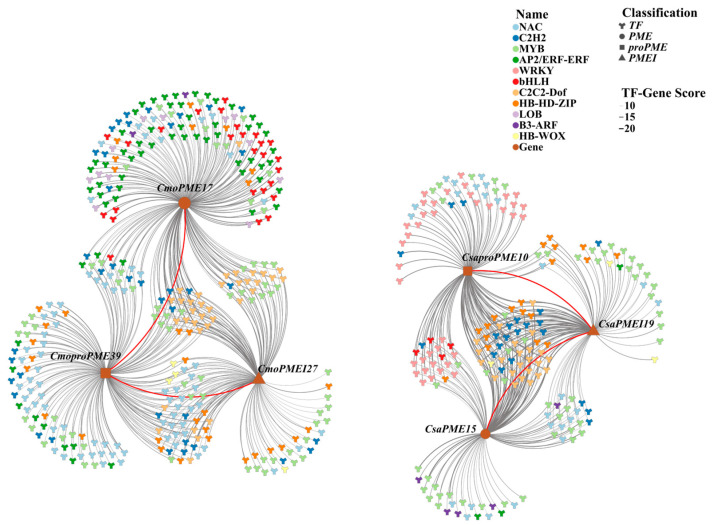
Regulatory network diagram for key members of the *PME/PMEI* gene families and key TFs in cucumber and pumpkin.

To delve deeper into the regulatory interactions between the pivotal transcription factors (TFs) and crucial members of the *PME*/*PMEI* gene families in cucumber and pumpkin, we selected the top 10 most abundant TF families regulating key *PME*/*PMEI* genes as well as TF families associated with graft union healing to establish an interactive regulatory network (Figure 16). The results revealed distinct differences in the TFs regulating the *PME*/*PMEI* gene families. Specifically, the AP2/ERF-ERF and bHLH TFs were predominantly involved in regulating the *PME* gene families, whereas LOB and the B3-ARF TFs exclusively participated in the regulation of type II *PMEs*. Furthermore, C2C2-Dof, C2H2, MYB, and HB-HD-ZIP TFs were also involved in regulating six key members of the *PME/PMEI* gene families, with C2C2-Dof TFs being the most abundant.

#### 2.8.3. Expression Analysis of Key Members of the PME/PMEI Gene Families and Dof TFs in Cucumber/Pumpkin Graft Healing Stages Under Different Light Intensity Modes

**Figure 17:**
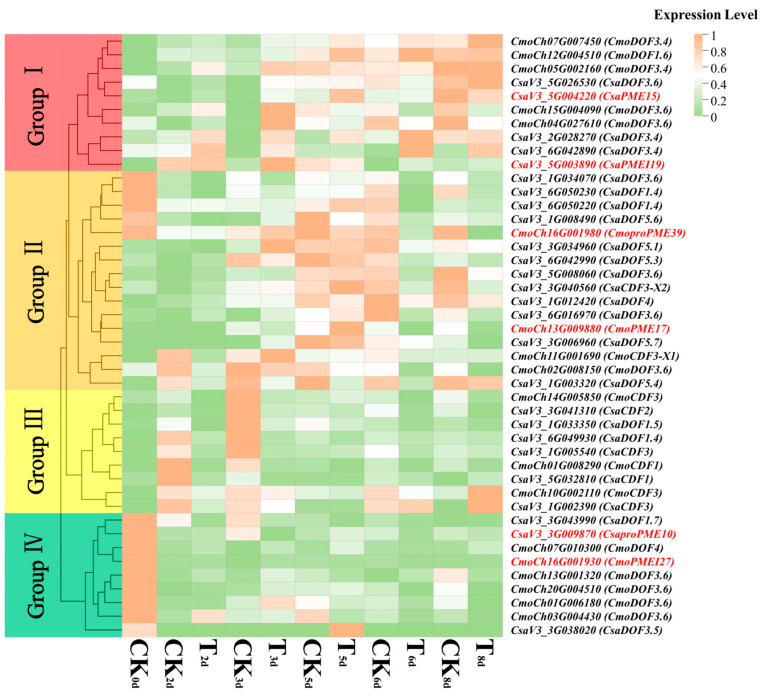
Expression analysis of key members of the *PME*/*PMEI* gene families and Dof TFs in the cucumber/pumpkin graft healing stages under different light intensity modes. The red font is the key members of the PME/PMEI family; the black font is the key members of the Dof TFs.

To delve deeper into the response modes of pivotal members of the *PME*/*PMEI* gene families and Dof transcription factors (TFs) in cucumber and pumpkin during the graft healing stage under varying light intensity modes, we identified C2C2-Dof as a crucial TF and conducted expression analysis alongside six key members of the *PME*/*PMEI* gene families (Figure 17). The results indicated that all of the pivotal members could be grouped into four groups. Specifically, *CsaPME15* and *CsaPMEI19* belonged to group I, which comprised eight Dof TF members exhibiting similar expression modes to these two genes. During the formation of the isolation layer (1–3 days post-grafting), their temporal expression profiles diverged. The *CsaPMEI19* expression levels rose over time, whereas *CsaPME15* expression decreased under the control (CK) treatment but increased under treatment T over time. Additionally, their expression levels at T_2d_ were elevated compared with those at CK_2d_. During the formation of callus (4–6 days post-grafting) and vascular bundles (7–9 days post-grafting), the *CsaPME15* expression levels increased over time in both processes. Conversely, the *CsaPMEI19* expression levels increased over time under the control (CK) treatment but decreased under T treatment in both processes. Furthermore, the expression level of *CsaPME15* was higher at T_5d_ compared with CK_5d_, and *CsaPMEI19* exhibited a higher expression at T_8d_ than at CK_8d_. *CmoproPME39* and *CmoPME17* were grouped in group II, along with 14 Dof TF members that displayed similar expression modes. Their expression levels declined during the formation of the isolation layer. Additionally, *CmoPME17* showed a higher expression at T_2d_ compared with CK_2d_. The expression of *CmoPME17* increased over time during callus and vascular bundle formation, whereas *CmoproPME39* increased under CK treatment but decreased under T treatment. Moreover, *CmoPME17* exhibited a higher expression at T_5d_ compared with CK_5d_. *CsaproPME10* and *CmoPMEI27* were grouped in group IV, together with seven Dof TF members that exhibited similar expression modes. During the formation of the isolation layer, their expression levels gradually decreased, while T treatment enhanced their expression. During callus formation, their temporal response characteristics were the opposite. Over time, *CsaproPME10* expression decreased under CK treatment but increased under T treatment. Additionally, the expression levels at T_5d_ were higher compared with those at CK_5d_. During vascular bundle formation, their temporal response characteristics remained consistent. Under CK treatment, the expression levels decreased over time, whereas under T treatment, they increased. Furthermore, the expression level of *CsaProPME10* was higher at CK_8d_ compared with T_8d_, whereas *CmoPMEI27* expression was lower at T_8d_.

**Figure 18:**
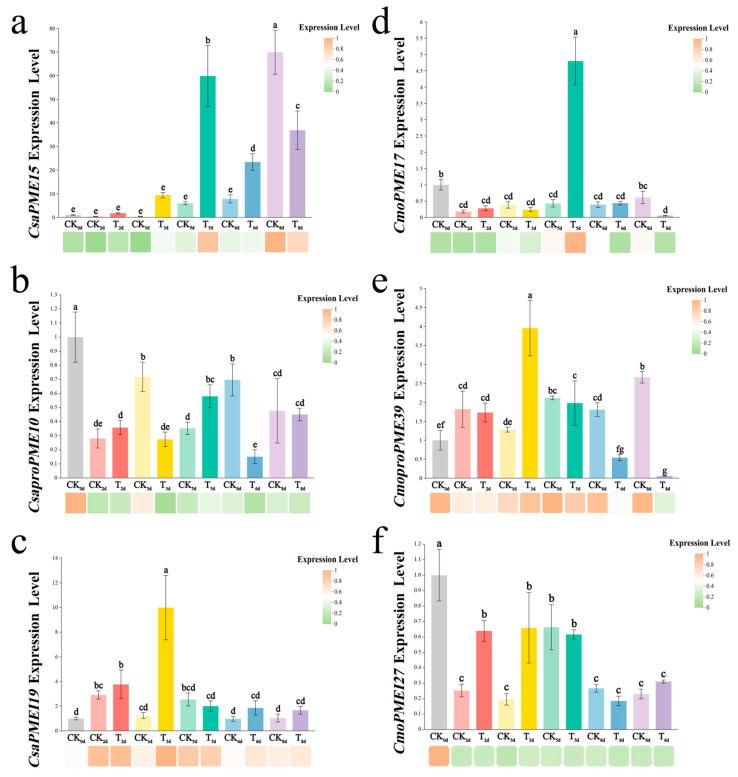
qRT-PCR validation. (**a**) *CsaPME15*; (**b**) *CsaproPME10*; (**c**) *CsaPMEI19*; (**d**) *CmoPME17*; (**e**) *CmoproPME39*; (**f**) *CmoPMEI27*. The bar chart and heatmap below represent the relative expression levels of six key members of the *PME*/*PMEI* gene families in the qRT-PCR and transcriptome, respectively. Lowercase letters indicate significance at the *p* < 0.05.

Subsequently, RT-qPCR validation was performed on six key members of the *PME/PMEI* gene family (Figure 18). The relative expression trends of each gene were generally in agreement with those obtained from transcriptome sequencing, suggesting that the sequencing results can be used to evaluate the upregulation and downregulation of the gene expression levels.

## 3. Discussion

### 3.1. Conserved Evolution and Functional Diversity of PME/PMEI in Cucumber and Pumpkin

Previous research endeavors have elucidated the *PME/PMEI* gene families across diverse species [21,22], underlining their pivotal roles in seed germination, growth, development, and stress adaptation mechanisms, with these functions being empirically validated in select organisms [23,24,25,27,31]. To procure a preliminary understanding into the functional attributes of the *PME/PMEI* families within cucumber and pumpkin, the present study undertook a comprehensive analysis based on the whole-genome sequences of these species. Consequently, 52 *CsaPME* family members (comprising 30 type I *CsaproPMEs* and 22 type II *CsaPMEs*), alongside 86 *CmoPME* family members (encompassing 48 type I *CmoproPMEs* and 38 type II *CmoPMEs*), were identified. Additionally, 33 *CsaPMEI* family members and 36 *CmpPMEI* family members were characterized. The physicochemical property analysis revealed a preponderance of hydrophilic *PME* family members in both cucumber and pumpkin, with the exception of six hydrophobic proteins, while no notable hydrophobicity was evident among the *PMEI* proteins. These findings mirror the physicochemical characteristics of *PME*/*PMEI* family members observed in other species [20,21,32]. Conservative sequence analysis further unveiled that both type I *proPMEs* and type II *PMEs* in cucumber and pumpkin possessed shared conserved sequences. Notably, type I *proPMEs* also harbored conserved *PMEI* sequences, underscoring the presence of evolutionary conserved motifs within these families. This is related to preventing premature demethylesterification during PME synthesis.

A collinearity analysis revealed that the expansion of the *PME*/*PMEI* family in cucumber and pumpkin could be primarily attributed to chromosomal segment duplication. Furthermore, the analysis of Ka/Ks values for *PME*/*PMEI* gene pairs within and between the cucumber and pumpkin groups consistently yielded values less than 1, indicative of purifying selection during their evolutionary trajectory [33,34]. These observations, coupled with insights from related studies, suggest a significant degree of conservation in the *PME*/*PMEI* genes, essential for maintaining their fundamental functions throughout evolution [21,35]. To delve deeper into the response mechanisms of *PME*/*PMEI* to environmental cues, hormonal signals, and other pertinent factors during plant growth and development and to establish a regulatory framework for *PME*/*PMEI*-related gene expression, a cis-acting element analysis was performed [36]. The results demonstrated that the *PME*/*PMEI* family members in both cucumber and pumpkin harbored elements associated with light, hormones, stress responses, and growth and developmental processes. Notably, while these genes shared numerous light-responsive elements and abscisic acid-responsive elements (ABREs) with cucumber, the *PME*/*PMEI* gene family in pumpkin also abundantly contained two types of methyl jasmonate (MeJA)-responsive elements: the CGTCA-motif and the TGACG-motif. This finding implies that *PME*/*PMEI* in both cucumber and pumpkin responds to growth, development, and stress through these cis-acting regulatory elements, with pumpkin exhibiting an enhanced capacity for stress tolerance.

### 3.2. Active Participation of Cucumber and Pumpkin PME/PMEI in Grafted Seedling Growth, Development, and Stress Response

The cell wall, functioning as an indispensable signaling response system, continuously monitors its status via the cell wall integrity (CWI) pathway. This sophisticated mechanism allows for the prompt response to cell wall damage (CWD) and the dynamic remodeling of the cell wall [37]. This intricate process involves the synthesis and degradation of both aged and newly synthesized cell wall components [38]. Among the constituents of the cell wall, pectin undergoes modulation by pectin methylesterase (PME) and its inhibitor (PMEI) through the adjustment of its degree of methylesterification. This regulation profoundly influences cell wall rigidity and plays a central role in maintaining cell wall integrity throughout plant growth, development, and stress responses. Studies have demonstrated that a high degree of methylesterified pectin is essential during rapid cell expansion, whereas the abundance of low methylesterified pectin in adjacent areas can impair cell wall extensibility, consequently affecting normal part differentiation in plants [39,40]. Furthermore, once cell growth ceases, PME acts to decrease the methylesterification level of pectin arranged in the cell outer wall, thereby enhancing cell wall rigidity [41,42].

In this investigation, Gene Ontology (GO) enrichment analysis revealed that *PME*/*PMEI* from cucumber and pumpkin are collaboratively involved in cell wall modification, pectin catabolism, and the negative regulation of catalytic activity. Notably, pumpkin *PME*/*PMEI* family members were specifically enriched in processes associated with stress responses. Expression analysis indicated that members of the *PME*/*PMEI* family actively participate in plant growth, development, and stress responses in both homologous- and heterologous-grafted seedlings of cucumber and pumpkin. Remarkably, part-specific differences in the expression of *PME*/*PMEI* family members were observed among the various grafted seedling combinations of cucumber and pumpkin, exerting distinct roles during leaf and root development. Furthermore, a greater number of *PME*/*PMEI* family members exhibited higher expression levels in the heterografted-cucumber seedlings compared with homografted seedlings. In the heterografted-pumpkin seedlings, part-specific expression was more pronounced, particularly in seedlings with pumpkin as the rootstock. These findings suggest that heterologous grafting favors cucumber growth and development, particularly leaf growth, while using pumpkin as the rootstock promotes root growth in heterografted seedlings through the responses of *PME*/*PMEI* family members. Additionally, under chilling stress, *PME*/*PMEI* family members in both homologous- and heterologous-grafted pumpkin seedlings responded more vigorously, indicating enhanced stress resistance in pumpkin. Concurrently, numerous *PME*/*PMEI* family members responded in the roots of the homologous- and heterologous-grafted seedlings of cucumber and pumpkin, with higher expression observed in the heterologous-grafted seedlings compared with the homologous-grafted ones. These results are consistent with previous studies on the mechanisms of *PME*/*PMEI* responses to growth and development processes and various stresses. For instance, *AtPME35* has been shown to enhance mechanical strength [41]; *CbPMEI1*, homologous to *AtPMEI13*, promotes *Arabidopsis* root growth by modulating the pectin methylesterification levels under low-temperature stress, thus striking a balance between vegetative growth and stress resistance [43]. Additionally, *AtPME41* collaboratively regulates ion leakage with brassinolide under chilling stress, enhancing stress resistance [44]. All of them further highlight the importance of *PME*/*PMEI* in plant growth, development, and stress adaptation.

In summary, members of the *PME*/*PMEI* family play pivotal roles in hormone signal transduction and stress response mechanisms throughout the growth and development of cucumber and pumpkin. Specifically, during periods of stress, the *PME*/*PMEI* family members in pumpkin undergo coordinated regulation by hormones, notably abscisic acid and methyl jasmonate (MeJA). By integrating the analyses of promoter cis-acting elements, Gene Ontology (GO) enrichment, and expression profiles, we can infer that pumpkin demonstrates enhanced stress resistance capabilities. Furthermore, heterologous grafting experiments, where cucumber serves as the scion and pumpkin as the rootstock, elicit responses from a broader spectrum of *PME*/*PMEI* family members. This grafting strategy facilitates optimal growth and development while also augmenting the overall stress resistance of the grafted seedlings.

### 3.3. Positive Responses of PME/PMEI in Cucumber and Pumpkin to Different Light Intensity Modes During Cucumber/Pumpkin Graft Healing

Recent research endeavors have delineated the cucumber/pumpkin graft healing process into three distinct stages: the isolation layer formation stage spanning 1–3 days, the callus formation stage encompassing 4–6 days, and the vascular bundle formation stage lasting from 7–9 days [45]. Delving deeper into histological insights, it is revealed that during the isolation layer formation stage, cells at the graft interface secrete pectin, fostering adhesion between the rootstock and scion while maintaining cell wall integrity [46]. As the isolation layer dissipates during the subsequent callus formation stage, pectic substances accumulate to form secondary plasmodesmata. These, coupled with callus responses such as the emergence of cell wall bulges, wall-adjacent bodies, vesicles, and multivesicular bodies, facilitate intercellular communication and cell recognition [47]. Pectin therefore plays a pivotal role in orchestrating callus responses during graft healing.

In this study, we investigated the response mechanisms of the *PME*/*PMEI* family to varying light modes during cucumber/pumpkin graft healing. In this study, when investigating the response mechanism of the *PME*/*PMEI* family to different light intensity modes during the grafting healing process of cucumber/pumpkin, it was found that compared with the traditional light intensity mode, the application of an optimal light intensity mode could induce the upregulation of *PME*/*PMEI* family members in both the stock and scion during the formation of the isolation layer. During the callus formation stage, it induced the upregulation of PME family members in the scion and inhibited the expression levels of *PMEI* family members in both the stock and scion. During the vascular bundle formation stage, it induced the upregulation of more PMEI in both the stock and scion. These findings suggest that when using the optimal light intensity mode, members of the *PME*/*PMEI* family in both the stock and scion actively participate in the dynamic remodeling of the cell wall and the formation of the isolation layer during the isolation layer formation stage. During the callus formation stage, it accelerates the formation of low-methylesterified pectin in a random catalytic manner by enhancing the efficiency of *PME* in the scion, thereby accelerating cell wall softening and promoting callus formation. During the vascular bundle formation stage, the expression level of PMEI is enhanced to promote the formation of high-methylesterified pectin, thereby accelerating the development of the vascular bundle. This mechanism aligns with the role of *AtPMEI5* [27] during graft healing.

In summary, the adoption of a higher light intensity mode can induce the binding of light-responsive elements and transcription factors to members of the *PME*/*PMEI* family in cucumber and pumpkin and regulate the level of pectin methylesterification through the interaction between the *PME* and *PMEI* families during the grafting healing process. This reduces the impact of mechanical damage and enhances the efficiency of grafting healing.

### 3.4. Transcription Factors in Cucumber and Pumpkin Influence Graft Union Healing in Cucurbits by Regulating Key Members of the PME/PMEI Family

Transcription factors (TFs) exert influence on the transcriptional regulation of downstream genes by specifically binding to target genes [48]. Previous studies have validated the involvement of TFs such as MYB, ERF, and Dof in regulating the *PME*/*PMEI* gene family and their impact on graft union healing in cucurbit crops [27,49,50]. For instance, *HCA2* and *TMO6* from the Dof TF family, *ERF115* from the ERF TF family, and *ANAC096* from the ANAC TF family regulate the degree of pectin methylesterification at the graft interface through interactions with *PMEI5*, thereby affecting the graft union healing efficiency.

Based on bioinformatics and expression analyses, this study screened six members of the *PME*/*PMEI* family in cucumber and pumpkin that exhibited specific expression modes related to grafting growth and development, chilling stress responses, and light responses during graft union healing: *CsaPME15*, *CsaproPME10*, *CsaPMEI19*, *CmoPME17*, *CmoproPME39*, and *CmoPMEI27*. During the construction of the TF regulatory network, it was found that TFs such as NAC, C2H2, MYB, ERF, WRKY, bHLH, Dof, HD-ZIP, LOB, ARF, and WOX are involved in regulating these six key members of the *PME/PMEI* family, with Dof TFs being the most prominent. Therefore, to further explore the temporal response characteristics of Dof TFs and key members of the *PME*/*PMEI* family to different light intensity modes during cucumber/pumpkin graft union healing and elucidate their mechanisms of action, transcriptome data analysis was conducted in this study. The results revealed that during cucumber/pumpkin graft union healing, key members of the *PME*/*PMEI* gene family and Dof TFs could be classified into four groups. In group I, *CsaPME15* and *CsaPMEI19* exhibited expression modes closest to those of *CsaDof3.6* and *CsaDof3.4*, respectively. In group II, *CmoproPME39* and *CmoPME17* showed expression modes closest to that of *CsaDof3.6*. In cluster IV, *CsaproPME10* and *CmoPMEI27* displayed similar expression trends to *CsaDof1.7* and *CmoDof4*, respectively. These findings suggest that during graft union healing, key members of *PME*/*PMEI* and Dof TFs in both the rootstock and scion regulate each other and collectively respond to different light intensity modes. During the formation stage of the isolation layer, the activation of key members of the *PME*/*PMEI* gene family is induced to accelerate the formation of the isolation layer. In the callus formation stage, the expression of key members of the *PME* family is upregulated, while the expression of key members of the *PMEI* family is downregulated, thereby inducing pectin methylesterase to catalyze the formation of low-methylesterified pectin in a random manner. This accelerates cell wall softening and promotes the proliferation and expansion of callus cells. During the vascular bundle formation stage, the expression of key members of the *PME* family is downregulated, while the expression of key members of the *PMEI* family is upregulated, inducing the formation of high-methylesterified pectin. Simultaneously, this ensures cell wall homeostasis and facilitates cell expansion during the process of vascular bundle formation, thereby affecting the process of graft union healing.

## 4. Materials and Methods

### 4.1. Plant Materials

In this study, the ‘Dongqing’ cucumber was utilized as the scion, while ‘Zhuangshi’ white-seeded pumpkin served as the rootstock for grafting. Both types of seeds were procured from Fuzhou Changyu Agricultural Development Co. Ltd. (Fuzhou, China). This grafting combination is one of the most commonly used combinations for grafted seedlings in Fujian Province.

### 4.2. Identification, Chromosomal Localization, and Protein Physicochemical Properties of PME/PMEI Family Members in Cucumber and Pumpkin

The reported *Arabidopsis PME*/*PMEI* family sequences were downloaded from the Arabidopsis Information Resource database (TAIR; https://www.arabidopsis.org) (accessed on 10 June 2024) [51]. The cucumber (Cucumber Chinese Long v3 Genome) and pumpkin (Cucurbita moschata Rifu v1 Genome) genomic data were downloaded from Cucurbit Genomics Database v2 (CuGenDBv2; http://cucurbitgenomics.org/v2/) (accessed on 10 June 2024) [52]. The candidate *PME*/*PMEI* family members in cucumber and pumpkin were screened using two approaches. Initially, the TBtools-II software (2.118) [53] was employed to conduct BLASTP analysis between the whole-genome protein sequences of cucumber and pumpkin and the *PME*/*PMEI* protein sequences of Arabidopsis, in order to identify potential *PME*/*PMEI* candidate genes in cucumber and pumpkin. Subsequently, the *PME*/*PMEI* family hidden Markov models (PF03283 and PF04043) were obtained from Pfam (https://www.ebi.ac.uk/interpro/entry/pfam/#table) (accessed on 12 June 2024) [54]. Then, HMMER online software (https://www.ebi.ac.uk/Tools/hmmer/search/phmmer) (accessed on 12 June 2024) [55], the Conserved Domain Database (https://ncbi.nlm.nih.gov/cdd) (accessed on 12 June 2024) [56], Pfam, and SMART (http://smart.embl-heidelberg.de) (accessed on 12 June 2024) [57] were used to reconfirm the pectinesterase and PMEI conserved structural domains of the potential *PME*/*PMEI* candidate genes. Finally, following the manual removal of duplicate and redundant sequences, a total of 52 cucumber *CsaPME* family members were obtained (comprising 30 type I *CsaproPMEs* and 22 type II *CsaPMEs*), along with 33 cucumber *CsaPMEI* family members. Similarly, 86 pumpkin *CmoPME* family members were identified, including 48 type I *CmoproPMEs* and 38 type II *CmoPMEs*, along with 36 pumpkin *CmoPMEI* family members. These genes were named sequentially according to their chromosomal locations.

Furthermore, the physicochemical properties and subcellular localization of the *PME*/*PMEI* family members from cucumber and pumpkin were predicted using ProtParam (https://web.expasy.org/protparam/) (accessed on 13 June 2024) [58] and WoLF PSORT (https://wolfpsort.hgc.jp/) (accessed on 13 June 2024) [59], respectively. The chromosomal localization was visualized using TBtools-II software according to the general guidelines.

### 4.3. Phylogenetic Analysis, Gene Structures, and Conserved Motif and Promoter Cis-Acting Element Predictions of PME/PMEI Family Members

The protein sequences of Arabidopsis thaliana, rice (*Oryza sativa*), wheat (*Triticum aestivum*), tomato (*Solanum lycopersicum*), and melon (*Cucumis melo*) were downloaded from TAIR and Phytozome (https://phytozome.jgi.doe.gov/pz/portal.html) (accessed on 13 June 2024) [60], respectively. The *PME*/*PMEI* gene protein sequences of cucumber (*Cucumis sativus*), pumpkin (*Cucurbita moschata*), *Arabidopsis thaliana*, rice, wheat, tomato, and melon were analyzed using MEGA11 software (version 11.0.13) [61]. Phylogenetic trees were constructed using the neighbor-joining (NJ) and maximum likelihood (ML) methods. Default program settings were applied, with 1000 bootstrap replicates for analysis. TBtools-II was employed to analyze gene structures, while CDD and MEME (https://meme-suite.org/meme/) (accessed on 13 June 2024) [62] were used to identify the conserved domains and motifs in the *PME*/*PMEI* proteins of cucumber and pumpkin. The upstream 2000 bp sequences of cucumber and pumpkin were analyzed for promoter cis-acting elements using PlantCare (https://bioinformatics.psb.ugent.be/webtools/plantcare/html/) (accessed on 13 June 2024) [63]. Finally, the aforementioned data were visualized using the Tree Visualization By One Table (tvBOT) online tool (https://www.chiplot.online/tvbot.html) (accessed on 16 June 2024) [64].

### 4.4. Analysis of Gene Collinearity and Gene Family Duplication Events in Cucumber, Pumpkin, and Arabidopsis Thaliana

The collinearity analysis was conducted using MCScanX software (https://github.com/wyp1125/MCScanX) (accessed on 13 June 2024) [65]. Visualization of the collinearity analysis and evolutionary analysis of selective pressure on the ortholog pairs were performed using TBtools-II. The visualization of the evolutionary analysis of selective pressure was created with Origin2021 (v9.8.0.200; OriginLab Corporation, Northampton, MA, USA).

### 4.5. GO Enrichment and Protein–Protein Interaction Network Analysis of the PME/PMEI Family Members in Cucumber and Pumpkin

GO enrichment analysis for cucumber and pumpkin was conducted using the Biomarker Cloud Analysis Platform (https://www.biocloud.net/fxpt/app) (accessed on 29 July 2024), and the results were visualized [66,67]. Protein–protein interaction (PPI) predictions for *PME*/*PMEI* proteins between Arabidopsis and cucumber were obtained from the STRING database (https://cn.string-db.org/) (accessed on 27 July 2024) [68]. The PPI predictions were then visualized using ChiPlot (https://www.chiplot.online/) (accessed on 29 June 2024).

### 4.6. Analysis of Expression Characteristics of the PME/PMEI Family Members in Cucumber and Pumpkin

Transcriptome sequencing data were screened from different vegetative organs of the cucumber and pumpkin homologous-grafted and heterologous-grafted seedlings (PRJNA55291445 [69]) based on the Cucurbitaceae database and NCBI database (https://www.ncbi.nlm.nih.gov/) (accessed on 10 June 2024). Additionally, transcriptome sequencing data were collected from different vegetative organs of cucumber and pumpkin homografted (cucumber/cucumber-grafted seedlings and pumpkin/pumpkin-grafted seedlings) and heterologous (cucumber/pumpkin and pumpkin/cucumber-grafted seedlings) grafted seedlings under chilling stress (PRJNA67308745 [69]). Data quality control and read counting were then performed using TBtools-II, followed by transcriptome data analysis based on the genome data of cucumber (Cucumber Chinese Long v3 Genome) and pumpkin (Cucurbita moschata Rifu v1 Genome).

### 4.7. Transcriptome Sequencing and qRT-PCR Analysis of PME/PMEI Family Members During Graft Healing Under Different Light Intensity Modes in Cucumber/Pumpkin-Grafted Seedlings

Grafting was conducted using an improved top-insertion method after the cucumber scion cotyledons were flattened and the pumpkin rootstocks had produced its first true leaf. After a 12-h recovery period at night, LED strips were used for illumination (the day of illumination initiation was recorded as the 1st day post-grafting). Two light intensity modes were set (Table 1, wavelength as shown in Figure S1), and other environmental factors were adjusted as specified in Table S2. Among them, CK represents the commonly used light intensity mode for the graft healing of cucumber/pumpkin in production, which involves applying light intensities of 0–50–100 μmol/(m^2^·s) on days 1–3, 4–6, and 7–9 after grafting, respectively. T, on the other hand, stands for the light intensity mode that is most conducive to cucumber/pumpkin graft healing, selected through preliminary experiments by observing the graft seedling morphology and measuring the chlorophyll fluorescence parameters. This mode involves applying light intensities of 50–100–150 μmol/(m^2^·s) on days 1–3, 4–6, and 7–9 after grafting, respectively [45]. Sampling was conducted on days 0, 2, 3, 5, 6, and 8 post-grafting. Samples collected on days 0 and 2 represented the first stage of graft healing, namely the formation of the isolation layer. Samples from the 3rd and 5th days represented the callus formation stage, while those from the 6th and 8th days represented the vascular bundle formation stage. Each treatment had three replicates, with 15 grafted seedlings per replicate. A 1-cm-long stem segment above and below the graft union was excised immediately, frozen in liquid nitrogen, and stored at −80 °C for subsequent transcriptome sequencing and RT-qPCR quantification of the key genes.

Three key family members were selected from each of the *PME* and *PMEI* gene families in cucumber and pumpkin for qRT-PCR analysis. Using actin as the reference gene, primers were designed using Primer 5.0 (Table S3). RNA extraction, reverse transcription, and qRT-PCR reactions were performed using the Polysaccharide and Polyphenolic Plant RNA Rapid Extraction Kit, HRbio™ III 1st Strand cDNA Synthesis SuperMix for qPCR (OneStep gDNA Removal) Kit, and the HRbio™ qPCR SYBR Green Master Mix (Low Rox Plus) Kit from Herui Biotech Co. Ltd (Fuzhou, Fujian, China). The reaction system consisted of 10.0 μL HRbio™ qPCR SYBR Green Master Mix (Low Rox Plus), 2 μL cDNA, 0.4 μL each of the forward and reverse primers (10 μM), and 7.2 μL ddH_2_O. Each gene in each treatment was repeated three times. The reaction involved an initial denaturation at 95 °C for 30 s, followed by 40 cycles of denaturation at 95 °C for 5 s and annealing/extension at 60 °C for 30 s. The melting curve was generated by heating at 95 °C for 15 s, 60 °C for 60 s, and finally extension at 95 °C for 15 s. The results were analyzed using the 2^−ΔΔCT^ method in ABI 7500 software (v.2.0.6) to determine the expression levels of the target genes. Significance analysis was conducted using Dunken’s method for multiple comparison tests (*p* < 0.05).

## 5. Conclusions

To elucidate the functions of the *PME*/*PMEI* gene families in cucumber and pumpkin during grafting wound healing, grafted seedling growth and development, and stress responses, this study identified 52 *CsaPME* family members (30 type I *CsaproPMEs* and 22 type II *CsaPMEs*) and 33 *CsaPMEI* family members in the cucumber genome as well as 86 *CmoPME* family members (48 type I *CmoproPMEs* and 38 type II *CmoPMEs*) and 36 *CmoPMEI* family members in the pumpkin genome. Based on phylogenetic and collinearity analyses, cucumber, pumpkin, and melon were determined to have closer genetic relationships. Additionally, various cis-elements related to light, hormones, and stress responses were identified in the *PME*/*PMEI* gene family members of cucumber and pumpkin. Subsequent expression analysis revealed a co-expression trend of *PME*/*PMEI* family members in cucumber and pumpkin during chilling stress responses, which were coordinately regulated by transcription factors such as NAC, MYB, and WRKY. These regulations altered the degree of cell wall methylesterification to enhance adaptability. Furthermore, the cucumber/pumpkin-grafted seedlings exhibited differential responses to different light intensity modes during wound healing. When using the optimal light intensity mode, members of the PME/PMEI family in both the stock and scion actively participated in the dynamic remodeling of the cell wall and the formation of the isolation layer during the isolation layer formation stage. During the callus formation stage, it accelerated the formation of low-methylesterified pectin in a random catalytic manner by enhancing the efficiency of *PME* in the scion, thereby accelerating cell wall softening and promoting callus formation. During the vascular bundle formation stage, it promoted the formation of vascular bundles by facilitating the formation of high-methylesterified pectin.

Based on these experiments, we further screened key *PME*/*PMEI* gene family members involved in responses to different light intensity modes during cucumber/pumpkin grafting healing: *CsaPME15*, *CsaproPME10*, *CsaPMEI19*, *CmoPME17*, *CmoproPME39*, and *CmoPMEI27*. Additionally, we identified key Dof transcription factors involved in the positive regulation of these six key *PME*/*PMEI* gene family members: *CsaDof3.6*, *CsaDof3.4*, *CsaDof1.7*, and *CmoDof4*. The results indicated that key Dof transcription factors in cucumber/pumpkin grafted seedlings respond to higher light intensities through signal transduction between the rootstock and scion, participating in the regulation of key genes in the *PME*/*PMEI* family, thus leading to the exhibition of expression characteristics: during the formation stage of the isolation layer, members of the *PME*/*PMEI* family in both the rootstock and scion are actively involved in the dynamic remodeling of the cell wall and the formation of the isolation layer, guided by the optimal light intensity mode. In the callus formation stage, by enhancing the activity of *PME* in the scion, the random catalysis of pectin methylesterase is accelerated, resulting in the formation of low-methylesterified pectin. This speeds up softening of the cell wall and promotes the proliferation and expansion of callus cells. During the vascular bundle formation stage, the process is facilitated by promoting the formation of high-methylesterified pectin through the regulation of *PME*/*PMEI* family members, which also ensures cell wall homeostasis and aids cell expansion. Consequently, these mechanisms affect the healing process of the graft union.

All of these findings contribute to a better understanding of the *PME*/*PMEI* families in cucumber and pumpkin. This provides a theoretical reference for the screening and creation of rootstock varieties with high stress resistance and high affinity. However, further in-depth research on the *PME*/*PMEI* gene families in cucumber and pumpkin is needed.

## Figures and Tables

**Table 1:** Light intensity modes during the graft healing period.

Mode	Light Intensity/μmol/(m^2^·s)		
	1–3 d	4–6 d	7–9 d
CK	0	50	100
T	50	100	150

## Data Availability

Data are contained within the article.

## Supplementary Materials

The following supporting information can be downloaded at: https://www.mdpi.com/article/10.3390/plants14091294/s1, Figure S1: LED spectral diagram; Table S1: Physicochemical property analysis of the *PME*/*PMEI* gene families in cucumber and pumpkin; Table S2: Additional environmental factors during graft healing period; Table S3: The primer sequences used in qRT-PCR.
