# Supplementary figures and images for: Genome-Wide Analyses and Expression Profiling of PME/PMEI Gene Families Reveal Their Relevance to Chilling Stress Response and Grafted Healing Efficiency in Cucumber/Pumpkin-Grafted Plants
Source: Plants (Basel). 2025 Apr 25;14(9):1294. doi: 10.3390/plants14091294 (PMC12073293; doi:10.3390/plants14091294)

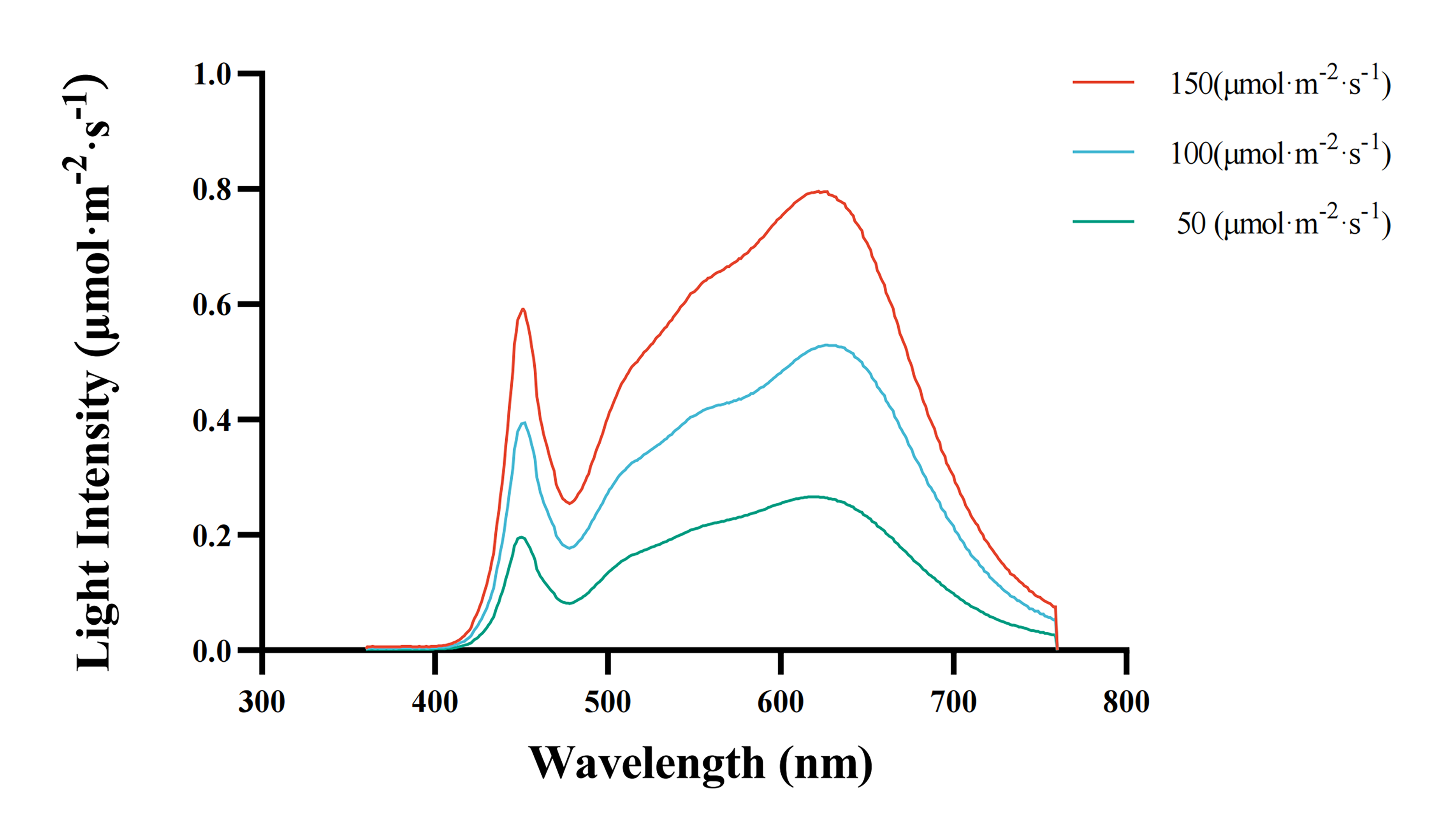

Supplement: Supplementary file 1 [file plants-14-01294-s001.zip › Supplementary Figure S1 LED spectral diagram.png]
